# Exploring the opinions and experiences of patients with generic substitution: a representative study of Polish society

**DOI:** 10.1007/s11096-014-0041-8

**Published:** 2014-11-27

**Authors:** Aleksandra Drozdowska, Tomasz Hermanowski

**Affiliations:** 1 Department of Pharmacoeconomics, Medical University of Warsaw, ul. Żwirki i Wigury 81, 02-091 Warsaw, Poland; 2 Department of Pharmacoeconomics, Polish Society of Health Economic, Medical University of Warsaw, ul. Żwirki i Wigury 81, 02-091 Warsaw, Poland

**Keywords:** Generic substitution, Generic medicines, Opinions, Patients, Poland

## Abstract

*Background* Generics have the potential to contain drug therapy costs; successful implementation of generic substitution policy largely depends on consumers’ willingness to choose generics. *Objective* This study aims to analyse the opinions, experiences and preferences of Polish patients towards generic medicines. *Setting* The study was performed in Poland. *Method* The survey was conducted in June 2013 by means of face-to-face interviews. Respondents were drawn from the general population according to a population structure. The study covered a representative sample of 1,000 Poles; the results can be generalized to apply to the Polish population at large. *Results* Fifty-two percent of respondents declared to be more often choosing generics, twenty-three percent did not have any specific preferences, and twenty-five percent were more willing to choose brand-name medicines. Past experience with cheaper generic medicines, secondary or lower education, low income and residence in specific regions of Poland were all significantly associated with an increased willingness to choose generics. Respondents’ attitudes towards generics were mostly influenced by the opinions of doctors and pharmacists. According to respondents, attitudes towards generics among doctors, pharmacists, family and friends, and in the mass media were mostly positive. There was no relationship between the preference of respondents for generics and factors such as their age, life stage, gender, household size or urban/rural locality. As a result of substituting a brand-name drug with its generic equivalent, 72 % of respondents reported that they had not noticed any difference in drug effectiveness; 21 % had experienced a reduced effectiveness of treatment or increased side effects at least once; and 7 % claimed the generic worked better. The majority of respondents who used cheaper substitutes claimed that generics represented good or very good quality. *Conclusion* The study demonstrates that, when choosing medicines, Poles rely mainly on the opinions of their doctors and pharmacists. Therefore, it is recommended that: (1) the option of using generics be promoted when writing prescriptions, and (2) the obligation on pharmacists to inform customers of their option to purchase generics be enforced.

## Impacts on practice

- Polish people generally prefer generic medicines over original medicines because they find them equally effective.
- A significant proportion of the Polish population have never used a generic substitute for an original drug. Therefore generic prescribing should be promoted, and pharmacists should meet their obligation to inform patients of cheaper medicines.
- Doctors and pharmacists were found to shape patients’ perception of generic medicines. Improved communication between patients and healthcare professionals around generics is recommended.

## Introduction

In 2010, the market share of generics in Poland was estimated at 40 % in value terms and 50 % in volume terms [1]. Drug prices in Poland are some of the lowest in Europe. Nonetheless, nearly 30 % of Polish patients do not use the prescriptions issued to them due to patient co-payment levels remaining very high [2], and only 18 % of medicines eligible for reimbursement being fully exempt from patient co-payment.

Increasing market share of generic medicines is seen as a remedy for the excessive financial strain caused by high drug spending and still insufficient access to medicines among some patient groups. Therefore, according to the Polish Ministry of Health, “the national drug policy should strive to rationalise pharmacotherapy expenditure to streamline access to cheap generic medicines” [3]. The escalating costs and affordability of medicines have become a burden for both governments and patients worldwide. One of the main methods of boosting the use of generics, and in the long term also cutting drug therapy costs, is generic substitution (GS).

Generic substitution policies have been implemented across the world to promote the use of low-priced generic medicines [4–7]. Under the Polish legal framework, in the case of reimbursable medicines, pharmacists are committed to substituting prescribed medicines for cheaper generic alternatives, unless the patient or the prescribing doctor opposes such a substitution [8]. Also, patients’ preferences have proved to be an obstacle in promoting generic prescription. From the patient perspective, the perceived quality of original medicines may be higher as compared to that of generic medicines [9].

## Aim of the study

The aims of this study were to evaluate consumers’ experience with generic medicines, find out whether consumers would be willing to use generic medicines, and investigate what are the factors influencing their choices. There was a need to study how often GS was the cause of altered or enhanced adverse effects in drug therapy. The perception of generics among family and friends, doctors, pharmacists, and the mass-media has also received attention. To the best of our knowledge, this is the first published study—that can be generalized to the whole Polish population—which explores factors determining patient choice of the type of prescribed medicines.

## Ethical approval

The Ethics Committee of the Medical University of Warsaw does not require consent, or give opinions, for this type of survey research.

## Method

### Representative sample

The study sample was a representative selection of 1,000 Poles from across Poland, offering the highest sample representativeness. A representative sample of the target population by definition allows researchers to draw valid conclusions about the studied phenomenon in the population at a predetermined significance level. In the present study, the prediction error is 3.16 % at 95 % statistical significance. This means, that the values determined in the study do not differ from population-wide values by more than 3.16 %.

The sample was randomly selected. Respondents were drawn from the population according to a population structure defined in the Electronic System of Population Register (PESEL) kept by the Polish Ministry of Internal Affairs. In accordance with art. 44 h, para. 2.2 of the Act on Registration of the Population and Identity Cards [PL: ustawa o ewidencji ludności i dowodach osobistych] dated 10 April 1974, the Ministry of the Interior made PESEL (national identification) numbers available to the authors for the purpose of conducting a public opinion poll. The PESEL numbers were chosen at random by custom designed software written by programmers at the Polish Ministry of Internal Affairs.

First the sample was stratified by nine regions and seven size categories of places of residence. Next, municipalities with probability proportional to the demographic structure of individual municipalities were randomly selected from each strata. In the next step, PESEL national identification numbers for further study participants were randomly selected at municipality level. The age and sex distribution was representative for each municipality. There were 601 respondents that turned out to be unavailable after the first selection due to: refusal—332, absence—182, others—87. For those individuals replacement persons were selected by quota sampling (representing the same controlled demographic characteristics as the primarily selected persons).

The sample then underwent post-stratification weighting adjustments to level off population distributions in terms of demographic and geographic parameters.

### Questionnaire

The questionnaire was tested for its face and content validity by four experts in public opinion research, and adjusted after pilot testing with 140 patients. The final questionnaire was composed of demographic questions as well as specific questions concerning experience and opinions of Poles in the area of GS. The patients’ attitudes to various aspects of GS were measured on a five-point Likert scale (in the analysis, we combined some answers). The survey was conducted in June 2013 by the means of face-to-face interviews. The term “generic substitution” was explained to each respondent before the interview has started. Respondents remained fully anonymous. Ethical approval was not required for this study.

### Statistical methods

SPSS Statistics v.21 (IBM Corp.) software was used for data analysis. Differences between groups were tested using the Mann–Whitney U test, while relationships between variables were examined using rho-Spearmans’s correlation—these non-parametric tests were used because of the non-Gaussian distribution of preferences towards medicines (tested using the Kolmogorov–Smirnov test).

## Results

### Preferences of respondents

More than half (52 %) of respondents declared to opt for generics if faced with a choice between brand name medicines and cheaper generic substitutes (23 % of respondents did not have any specific preferences, 25 % of respondents were more willing to choose original medicines), as shown in Table 1.

The level of education statistically differentiated the choice of respondents: those with a university degree opted significantly often for a brand name drug (U = 57,553, *p* = 0.001).

Net income per household was another factor which significantly differentiated respondent choices. Individuals with up to PLN 2,999 net income per household (USD 999.6) significantly more often preferred cheaper generic substitutes as compared to respondents with PLN 3,000–4,499 (USD 1,000–1,499.6) (U = 60,008; *p* = 0,048) and >PLN 4,500 (1,500 USD) (U = 56,684.5; *p* = 0,001) net income per household.

Regions of Poland also proved significant in the context of generic versus brand name drug choices made by respondents. Generics were found more popular all across Poland. In south-west Poland, preferences towards generics were demonstrated to be higher than in south-east Poland (U = 21,236, *p* = 0.04). No statistically significant differences were found between other regions.

Other analysed factors: sex, age, stage of life, household size, and locality (rural vs. urban area) did not differentiate the respondents to a statistically significant extent in terms (*p* > 0.05).

### Quality assessment of cheaper generic medicines

Forty-two percent of respondents claimed they had never used a generic substitute for an original drug. The majority of those who used cheaper substitutes claimed that generics represented good or very good quality (81 %). Eighteen percent who had had previous experience with GS expressed a negative opinion on generic quality (which they claimed were bad or very bad) (Table 2).

**Table 1:** Study population characteristics in terms of the preferences for GS (trichotomised as having preferences towards generics, towards brand name medicines, or no preferences, n = 1,000)

Sociodemographic characteristic	Total (n)	Patients who prefer generics, n (%)	Patients who prefer brand name medicines, n (%)	Patients who have no preferences, n (%)
*Gender*				
Female	524	293 (56 %)	131 (25 %)	99 (19 %)
Male	477	229 (48 %)	124 (26 %)	128 (27 %)
*Age*				
15–29 year	274	125 (46 %)	81 (30 %)	68 (25 %)
30–39 year	171	84 (49 %)	46 (27 %)	41 (24 %)
40–59 year	333	180 (54 %)	70 (21 %)	83 (25 %)
60 year and over	223	133 (59 %)	29 (13 %)	64 (27 %)
*Education*				
Secondary or lower	850	455 (53 %)	206 (24 %)	189 (22 %)
University	150	69 (46 %)	59 (39 %)	23 (15 %)
*Household size*				
Single	86	48 (56 %)	21 (25 %)	17 (20 %)
2 persons	162	88 (55 %)	42 (26 %)	32 (20 %)
3 persons	208	96 (46 %)	52 (25 %)	60 (29 %)
4 persons	252	134 (53 %)	55 (22 %)	63 (25 %)
5 persons and more	292	156 (54 %)	81 (28 %)	55 (19 %)
*Stage of life*				
Students, single, live with parents	98	37 (38 %)	23 (23 %)	38 (39 %)
Professionally active, single, live with parents	73	35 (48 %)	17 (23 %)	22 (30 %)
Young adults, no kids, own household	23	11 (48 %)	4 (17 %)	8 (35 %)
Family with kids	535	289 (54 %)	112 (21 %)	134 (25 %)
Older families, professionally active, no kids at home	102	51 (50 %)	29 (28 %)	22 (22 %)
Older families, non-working, no kids at home	163	96 (59 %)	46 (28 %)	21 (13 %)
*Total household income (net, per month)*				
Up to PLN 2,999 PLN (USD 999.6)	427	252 (59 %)	92 (22 %)	83 (20 %)
PLN 3,000–4,499 PLN (USD 1,000–1,499.6)	283	142 (50 %)	76 (27 %)	65 (23 %)
PLN 4,500 (USD 1,500) and more	290	129 (45 %)	83 (29 %)	78 (27 %)
*Locality*				
Rural area	386	195 (51 %)	115 (30 %)	76 (20 %)
Urban area	614	322 (53 %)	140 (23 %)	152 (25 %)
*Region of Poland*				
Central	202	106 (53 %)	54 (27 %)	42 (21 %)
North-east	127	70 (56 %)	36 (29 %)	20 (16 %)
North-west	244	118 (48 %)	47 (19 %)	78 (32 %)
South-west	253	141 (55 %)	51 (20 %)	61 (24 %)
South-east	174	87 (50 %)	63 (36 %)	24 (14 %)

**Table 2:** Perception of generics based on respondents’ personal experience (n = 580)

Experience regarding the cheaper equivalents of original medicines^a^	n	%
Patients who have used generic medicines and think that they are usually of good quality	355	61
Patients who have used generic medicines and think that they are usually of very good quality.	117	20
Patients who have used generic medicines and think that they are usually of bad quality	48	8
Patients who have used generic medicines and think that they are usually of very bad quality	60	10

^a^Respondents who have never used a cheaper generic substitute for an original drug were excluded from the analysis

Taking into account the respondents who had had experience with generic medicines: the better the respondents assessed the quality of generics, the more willing they were to choose generics instead of brand name medicines (Spearman’s rho = 0.3 and *p* < 0.001).

### Experiences with generic substitution

The opinion of respondents on the quality of generics they had used (Table 2) was not shown in comparison with brand name medicines. Contrariwise, their experience with GS (Table 3) is explained with reference to their previous experience with brand name medicines.

**Table 3:** Respondents’ personal experience with GS (n = 560)

Sociodemographic characteristic	Total^a^ (n)	Patients who have at least once experienced lower efficacy following generic substitution, n (%)	Patients who have at least once experienced aggravation of adverse effects following generic substitution, n (%)	Patients who did not notice any difference—a generic substitute has always been as effective as an original drug, n (%)	Patients who claimed that a generic substitute was sometimes more effective than its original equivalent, n (%)
*Gender*					
Male	220	33 (15 %)	24 (11 %)	149 (67 %)	14 (7 %)
Female	340	42 (12 %)	21 (6 %)	256 (75 %)	21 (6 %)
*Age*					
15–29 year	118	11 (9 %)	8 (7 %)	92 (78 %)	7 (6 %)
30–39 year	107	19 (18 %)	4 (4 %)	80 (75 %)	4 (4 %)
40–59 year	186	25 (13 %)	19 (10 %)	128 (69 %)	15 (8 %)
60 year and over	149	21 (14 %)	13 (9 %)	105 (70 %)	10 (7 %)
*Education*					
Secondary or lower	478	65 (14 %)	39 (8 %)	341 (71 %)	33 (7 %)
University	82	8 (15 %)	5 (8 %)	66 (71 %)	3 (6 %)
*Stage of life*					
Students, single, live with parents	34	3 (9 %)	1 (3 %)	30 (89 %)	0 (0 %)
Professionally active, single, live with parents	24	2 (9 %)	3 (13 %)	15 (63 %)	4 (16 %)
Young adults, no kids, own household	15	1 (3 %)	2 (14 %)	12 (79 %)	1 (5 %)
Family with kids	319	40 (13 %)	25 (8 %)	230 (72 %)	24 (8 %)
Older families, professionally active, no kids at home	57	7 (13 %)	5 (9 %)	41 (71 %)	4 (7 %)
Older families, non-working, no kids at home	111	21 (19 %)	6 (6 %)	78 (71 %)	5 (4 %)
*Total household income (net, per month)*					
Up to PLN 2,999 PLN (USD 999.6)	250	39 (15 %)	20 (8 %)	176 (71 %)	15 (6 %)
PLN 3,000–4,499 PLN (USD 1,000–1,499.6)	139	17 (12 %)	3 (2 %)	110 (80 %)	9 (6 %)
PLN 4,500 (USD 1,500) and more	171	17 (10 %)	17 (10 %)	122 (71 %)	15 (8 %)
*Locality*					
Rural area	193	31 (16 %)	19 (10 %)	131 (68 %)	12 (6 %)
Urban area	367	43 (12 %)	24 (7 %)	276 (75 %)	24 (7 %)
*Region of Poland*					
Central	120	14 (12 %)	10 (8 %)	86 (72 %)	10 (8 %)
North-east	64	8 (13 %)	1 (2 %)	50 (78 %)	5 (8 %)
North-west	157	22 (14 %)	24 (15 %)	104 (66 %)	7 (4 %)
South-west	140	20 (14 %)	5 (4 %)	103 (74 %)	12 (9 %)
South-east	79	10 (13 %)	3 (4 %)	64 (81 %)	2 (3 %)

^a^Respondents who did not have comparison between original and generic medicines were excluded from the analysis

Many respondents declared to have had no comparison between generic and original medicines (44 %). Among respondents who had had an experience with original medicines and cheaper equivalents in the past, 72 % did not observe any difference (both worked well), and 7 % claimed the generic worked better. 14 and 7 % of respondents reported experiencing lower efficacy and aggravation of adverse effects following GS, respectively, at least once. The analysis that was performed by using the Chi square test did not show any significant differences in the experience regarding the effectiveness of generic medicines (as compared to brand name medicines) in relation to the socio-demographic variables: gender, age, stage of life, income of a household, and place of residence—region and rural versus urban area. (*p* < 0.05) (Table 3).

### Opinions of different groups and their effect on respondents’ attitudes towards generics

**Fig. 1:**
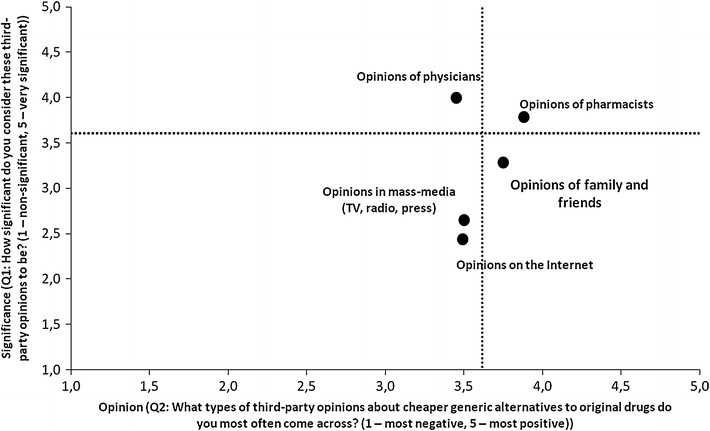
Opinions of different groups and their effect on respondents’ attitudes towards generics

The survey also examined which sources of information about generic medicines are considered by Poles to be crucial (in a 5-point scale ranging from 1—non-significant, to 5—significant) (Fig. 1). Respondents’ attitudes towards generics were found to be: mostly influenced by the opinions of doctors (4 on a 5-point scale); pharmacists (3.78 on a 5-point scale); to a lesser extent influenced by family and friends (3.28 on a 5-point scale); less influenced by the opinions presented in the mass-media (TV, radio, press—2.66 on a 5-point scale); and least by the Internet (2.43 on a 5-point scale).

According to respondents, attitudes towards generics among doctors, pharmacists, family and friends, and in the mass-media (TV, radio, press, and the Internet) were rather positive (an average of 3.6 on a 5-point scale).

## Discussion

The results of this study clearly demonstrate that Poles generally prefer generic medicines over original medicines and believe that generics are of good quality. The majority of respondents with prior experience of GS claimed that the generic substitute and its brand name equivalent proved to be equally effective. Only 14 % of respondents reported experiencing lower efficacy and 7 % reported an exacerbation of adverse effects following GS, both on at least one occasion. Negative experiences associated with the effectiveness of generic medicines (as compared to brand name medicines) did not statistically vary with respect to socio-demographic variables, i.e. gender, age, household income, place of residence, etc. Other studies also did not find a significant relationship between negative experiences associated with using generic medicines—such as overall dissatisfaction, more side-effects and lower effectiveness (as compared to brand name medicines)—and socio-demographic variables e.g. personal data [9]. Negative experience with generic substitutes, attributed to a lack of efficacy or adverse events, have also been reported in many earlier studies [9–12]. However, Himmel et al. [10] concluded that, since adverse effects caused by generics are inconsistent with pharmacological findings [13, 14], the experience of lower efficacy or non-specific side effects of generic medicines may be, at least among some patients, a nocebo phenomenon [15], (or an “adverse placebo effect”).

In the present study, 42 % of respondents claimed that they had never used a cheaper generic substitute for an original drug. This can to some extent be explained by another study by Jaźwińska-Tarnawska [16] in which almost 50 % of Polish patients did not ask for a cheaper substitute, and pharmacists did not inform patients about their GS options.

This study also revealed that Poles do take into account the opinions of doctors and pharmacists, and that these healthcare professionals typically express positive attitudes towards generics. There are a number of other studies in which doctors and pharmacists were shown to have heavily influenced the choice of patients between brand name or generic medicines [17–21]. In other studies, many patients were found to have readily accepted generics if they had been prescribed or approved by doctors [10, 11, 22, 23] or recommended by pharmacists [9, 24].

In this study, there was no statistically significant relationship between the age of respondents and their choice of generic versus original medicines. There is no consensus in the literature regarding the differentiation of age on the preferences of patients between generic and brand name medicines [21, 24].

There were essentially no statistically significant differences between respondents of different genders in terms of their attitude towards GS. Other studies were in line with our findings. [9, 23, 25].

Net monthly income per household did statistically differentiate the respondents’ choices. Respondents with the highest income per household (over PLN 4,500/USD 1,500) were significantly more likely to opt for brand name medicines compared to those on the lowest income (PLN 2,999/USD 999.60). In this sense, it can be hypothesized that drug price is the basic criterion, which determines the generic versus brand name drug choices made by poorer patients.

In the study performed by Håkonsen et al. [26] in 2012, which addressed the question of patient perspectives on GS, patients with higher educational levels were found to be consistently more likely to hold positive attitudes towards GS. This conclusion is inconsistent with the results of our study, which showed that respondents with a university education were significantly more likely to prefer original medicines compared to respondents without degrees. A study by Professor J Czapiński demonstrated that individuals with university degree earned 42 % more than those with a secondary school education [27]. This could point to the income level as being the main reason why university graduates were more likely to prefer brand name products over generic substitutes. We may infer that in Poland brand name medicines are only affordable to the wealthiest and those who are relatively poor prefer generics. However, this does not provide an in-depth explanation as to why the attitude of Polish respondents holding a university degree towards generics is so different compared with other countries. This question needs to be addressed in future research. In addition, as our survey focused on general preferences for generics, further research should be more detailed and could investigate, for example, the willingness of patients to switch depending on the type of generic and therapeutic group.

## Conclusion

While most Polish patients appear to have a positive attitude towards generics, a sizable minority prefers original brand name medicines. This study demonstrated that doctors and pharmacists can influence the perception of patients towards generic medicines. We thus propose that the following measures to increase the uptake of generics in Poland: improved doctor-patient communication; the promotion of GS at the prescribing stage and enforcing more effectively the obligation of pharmacists to inform patients of cheaper medicines.
